# Malignancy in toxic thyroid adenoma: Revisiting risk assessment and identifying predictors

**DOI:** 10.1007/s00405-026-10083-5

**Published:** 2026-03-05

**Authors:** Murat Calapkulu, Ilknur Ozturk Unsal, Muhammed Erkam Sencar, Derya Cayir, Davut Sakiz, Merve Tekinyildiz, Murat Bugra Görgülü, Ömer Bayir, Tugba Taskin Turkmenoglu, Mustafa Ozbek, Erman Cakal

**Affiliations:** 1 Department of Endocrinology and Metabolism, Ankara Etlik City Hospital, Ankara, Turkey; 2 Department of Endocrinology and Metabolism, Ankara Medicana International Hospital, Ankara, Turkey; 3 Department of Endocrinology and Metabolism, Atilim University, Ankara, Turkey; 4 Department of Nuclear Medicine, Ankara Etlik City Hospital, Ankara, Turkey; 5 Department of Endocrinology and Metabolism, Mardin Artuklu University, Mardin, Turkey; 6 Department of Nephrology, University of Health Sciences, Ankara Training and Research Hospital, Ankara, Turkey; 7 Department of Otolaryngology and Head & Neck Surgery, Ankara Etlik City Hospital, Ankara, Turkey; 8 Department of Pathology, Ankara Etlik City Hospital, Ankara, Turkey

**Keywords:** Toxic adenoma, Thyroid cancer, fT4/fT3 ratio, EU-TIRADS classification

## Abstract

**Background:**

Toxic adenomas have traditionally been considered benign due to chronic TSH suppression, which is believed to inhibit thyroid tumorigenesis. However, emerging data challenge this dogma, reporting non-negligible malignancy rates even in toxic adenoma. This study aimed to assess thyroid cancer frequency and characteristics in surgically selected patients with toxic adenomas and to compare outcomes with propensity score–matched, surgically treated non-functioning nodules.

**Methods:**

This retrospective, cross-sectional study included 204 surgically treated patients at a tertiary referral center, comprising 102 surgically selected toxic adenomas and 102 propensity score–matched, surgically treated non-functioning nodules. Clinical, biochemical, sonographic, scintigraphic, and histopathological data were analyzed. Multivariate logistic regression analysis was used to identify independent predictors of malignancy among toxic adenomas.

**Results:**

In this surgically selected cohort, the malignancy rate was 16.7% for toxic adenomas and 40.2% for non-functioning nodules (*p* < 0.001). Papillary thyroid carcinoma comprised 82.4% of all cases, making it the leading histotype (82.4%). Among toxic nodules, higher fT4/fT3 ratio (cut-off:2.58, sensitivity:93.3%, specificity:54.2%) and European Thyroid Imaging and Reporting Data System categories 4–5 were independent predictors of malignancy. No significant differences were found between groups in terms of tumor size, invasion, American Thyroid Association risk stratification, or 5-year response rates.

**Conclusion:**

Among surgically treated patients, the observed malignancy rate in toxic adenomas appears to be higher than traditionally expected. Elevated fT4/fT3 ratio and suspicious ultrasound features warrant closer evaluation. These findings support using ultrasound and biochemical markers in risk assessment of all thyroid nodules, regardless of functional status.

## Introduction

Toxic adenomas are autonomously functioning thyroid nodules that have traditionally been regarded as benign lesions. The chronically suppressed thyroid-stimulating hormone (TSH) levels observed in these patients are thought to limit thyroid cell proliferation, thereby potentially exerting a protective effect against malignant transformation 1. Based on this theoretical framework and the low risk of malignancy, cytological evaluation of hyperfunctioning thyroid nodules is not routinely recommended by major clinical guidelines, including those of the American Thyroid Association (ATA) and the European Thyroid Association (ETA) 2, 3.

However, recent studies have reported that the prevalence of malignancy in toxic nodular thyroid disease, while generally lower than in non-toxic nodules, may be higher than previously assumed 4–6. In a multicenter study by Smith et al., the malignancy rate in patients with toxic nodular goiter was found to be 18.3%, while Tam et al. reported a similar rate of 19% [4, 5]. Dirikoç et al. reported malignancy rates of 23.7% in toxic nodular goiter and 24.9% in toxic multinodular goiter (TMNG), both of which significantly exceed previously published estimates 6. These findings necessitate a re-evaluation of the long-standing assumption that hyperthyroidism confers protection against thyroid cancer.

This study aimed to assess whether surgically selected toxic adenomas harbor a clinically relevant risk of malignancy, to identify independent biochemical and ultrasonographic predictors, and to compare oncologic outcomes with those of surgically treated non-functioning nodules.

## Materials and methods

### Study design and patient selection

**Fig. 1:**
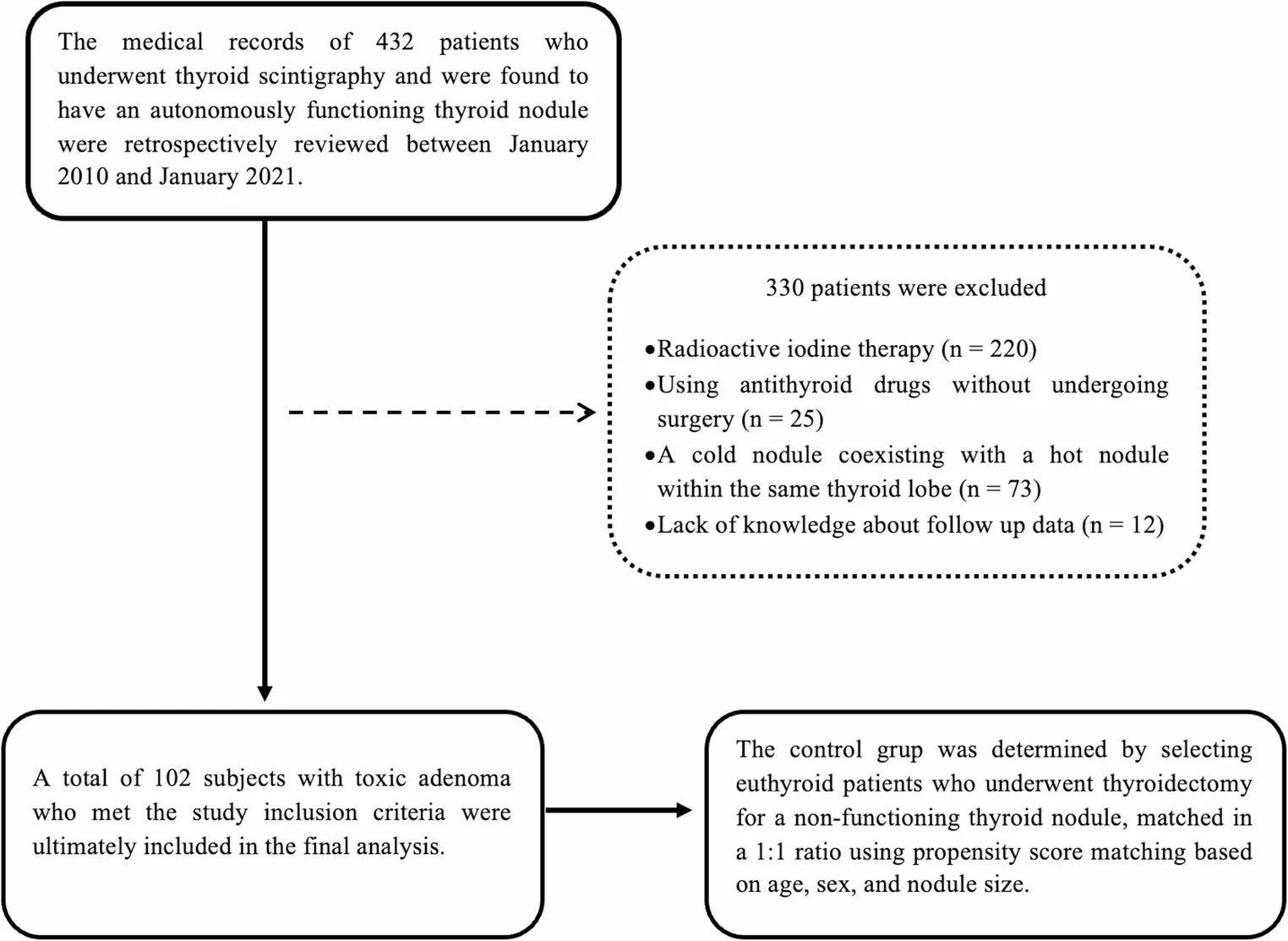
Flowchart of patient selection and inclusion process

This was a retrospective, cross-sectional study conducted at the University of Health Sciences, Diskapı Yildirim Beyazit Training and Research Hospital, a high-volume tertiary referral center located in Ankara, Turkey. The study was conducted in accordance with the principles of the Declaration of Helsinki and was approved by the local ethics committee of the University of Health Sciences Dışkapı Yıldırım Beyazıt Training and Research Hospital (06.06.2022–139/11). Due to the retrospective nature of the study and the use of anonymized patient data, informed consent was not obtained from the participants. Demographic characteristics, pathological features, and follow-up data of the patients were retrospectively collected from both electronic and archived medical records. First, the medical records of 432 patients diagnosed with toxic adenoma between January 2010 and January 2021 were retrospectively reviewed. Exclusion criteria were: age under 18 years, history of head and neck surgery, history of radiotherapy, absence of surgical intervention for toxic adenoma/TMNG, incomplete follow-up data, other causes of thyrotoxicosis such as Graves’ disease or subacute thyroiditis, and the presence of a cold nodule in the same lobe as the toxic adenoma. Only surgically treated patients were included in the study, as histopathological examination of surgical specimens represents the gold standard for the definitive diagnosis of thyroid malignancy. Furthermore, one of the primary aims of the study was to compare clinicopathological characteristics and oncologic outcomes, including tumor features, ATA risk stratification, and long-term treatment response, which can be reliably assessed only in patients with complete surgical pathology and follow-up data. The study included 102 patients with toxic adenoma who had a solitary toxic adenoma in one lobe and underwent surgery for this reason. To construct a statistically comparable control group, a cohort of 1205 patients with non-functioning thyroid nodules who underwent thyroidectomy or lobectomy was initially reviewed. After excluding patients with Graves’ disease, toxic nodular goiter, and TMNG, eligible cases were assessed for matching. To reduce potential selection bias and ensure baseline equivalence between groups, propensity score matching (PSM) was applied. Propensity scores were calculated using a multivariable logistic regression model that included the following covariates: age, sex, and nodule size (longest diameter), all of which are known to influence thyroid malignancy risk. Matching was performed using the nearest-neighbor method without replacement in a 1:1 ratio, ensuring close matches based on the logit of the propensity score. Using this approach, 102 euthyroid patients with non-functioning solitary nodules were matched to the toxic adenoma cohort. Balance between groups was confirmed by statistical tests showing no significant differences in key baseline characteristics. The patient selection flowchart is presented in Fig. 1.

### Clinical and biochemical measurements

All included patients underwent detailed clinical evaluation at presentation, including assessment of symptoms related to hyperthyroidism, personal and family history of thyroid disease, and prior exposure to ionizing radiation. The diagnosis of toxic adenoma was established based on: (1) serum TSH levels below the reference range with either elevated or normal free triiodothyronine (fT3) or free thyroxine (fT4); (2) the presence of a thyroid nodule on ultrasonography (USG); and (3) a hyperfunctioning nodule on Tc-99 m pertechnetate scintigraphy showing suppressed uptake in extranodular tissue. Biochemical parameters included serum thyroid-stimulating hormone (TSH), serum thyroglobulin (Tg), anti-thyroglobulin antibody (anti-Tg Ab), free triiodothyronine (fT3), and free thyroxine (fT4). Thyroid function tests were measured using chemiluminescent immunoassays (Beckman Coulter, USA). Reference ranges were: TSH 0.38–5.33 µIU/mL, fT3 2.28–4 pg/mL, fT4 0.60–1.25 ng/dL.

### Sonographic and scintigraphic evaluations

All patients underwent high-resolution thyroid ultrasonography (USG) performed by experienced endocrinologist using a 13-MHz linear transducer (Hitachi HI VISION Preirus, Japan). Sonographic features such as nodule size, margins, echogenicity, calcifications, vascularity, and presence of peripheral halo were recorded. Nodules were classified according to the European Thyroid Imaging and Reporting Data System (EU-TIRADS). Technetium-99 m (Tc-99 m) pertechnetate thyroid scintigraphy was performed 15–20 min after intravenous injection of 5 mCi ± 1 (185 MBq ± 37) Tc-99 m pertechnetate. Images were acquired using a gamma camera with a pinhole collimator (Siemens ecam-signature; Siemens, Hoffmann Estates, IL, USA) in a 256 × 256 matrix with a 1.78 zoom. The energy window was maintained at 20%, with the photopeak centred at 140 keV. Before imaging, the patients were sometimes given water to eliminate oesophageal activity. Images were obtained from the anterior, right anterior oblique, and left anterior oblique projections. Over 100 counts were performed. A toxic nodule or multiple toxic nodules showed focally/multifocally increased uptake (hot areas), and uptake in normal thyroid tissue was more or less suppressed on thyroid scintigraphy.

### Fine-needle aspiration biopsy and surgical indication

Ultrasound-guided fine-needle aspiration biopsy (FNAB) was performed for nodules that met the EU-TIRADS guideline criteria for cytological evaluation, including suspicious sonographic features 3. Cytological results were categorized using the Bethesda System for Reporting Thyroid Cytopathology (categories I–VI) 7. Surgical indications were defined according to the functional status of the nodules. In patients with toxic adenomas, surgery was performed for cytologically malignant or suspicious nodules (Bethesda V–VI), indeterminate cytology (Bethesda III–IV) or repeatedly non-diagnostic cytology (Bethesda I) accompanied by suspicious clinical or ultrasonographic features, resistance or intolerance to medical therapy, compressive symptoms, or patient preference after achieving euthyroidism. In contrast, patients with non-functioning thyroid nodules underwent surgery for malignant or suspicious cytology (Bethesda V–VI), indeterminate (Bethesda III–IV) or repeatedly non-diagnostic cytology (Bethesda I) with high-risk clinical or ultrasonographic features, documented nodule growth, compressive symptoms, or patient preference.

### Follow-up assessments

All patients were monitored postoperatively according to the ATA guidelines, with risk stratification performed after histopathologic diagnosis 8. Tg, anti-Tg Ab, and TSH levels were monitored at regular intervals during follow-up. High-resolution neck ultrasonography was conducted every 3–6 months based on the initial risk category and response to therapy.

### Statistical analysis

All statistical analyses were performed using IBM SPSS Statistics for Windows, version 27.0 (IBM Corp., Armonk, NY, USA). The distribution of numerical variables was assessed using the Shapiro-Wilk test and visual inspection of histograms. Propensity score matching was used solely for the selection of a control group with comparable age, sex, and nodule size to the toxic adenoma cohort. Continuous variables were expressed as mean ± standard deviation (SD) for normally distributed data, and as median (interquartile range, IQR) for non-normally distributed data. Categorical variables were presented as frequencies and percentages. Comparisons between groups (toxic vs. non-functioning nodules, benign vs. malignant histology) were made using the independent samples t-test or the Mann–Whitney U test for continuous variables, depending on distribution normality. Chi-square test or Fisher’s exact test was used for categorical variables, as appropriate. The association between clinical, biochemical, and imaging findings and malignancy was evaluated using univariate analysis, and variables with *p* < 0.250 were entered into a multivariate logistic regression model to identify independent predictors of malignancy. Results were reported as odds ratios (ORs) with 95% confidence intervals (CIs). To determine an optimal threshold value for a continuous variable in relation to malignancy risk, a receiver operating characteristic (ROC) curve analysis was performed. The area under the curve (AUC), 95% confidence interval, and corresponding sensitivity and specificity values were calculated. A p-value < 0.05 was considered statistically significant for all analyses.

## Results

A total of 204 patients who underwent thyroidectomy were included, comprising 102 cases of toxic adenoma and 102 cases of non-functioning thyroid nodules. Table 1 presents the complete demographic and clinical characteristics of patients, comparing those with toxic adenomas to those with non-functioning thyroid nodules. There were no significant differences between the groups in terms of age (*p* = 0.590), gender distribution (*p* = 0.876), or nodule size (*p* = 0.605). TSH levels were significantly lower in the toxic adenoma group (*p* < 0.001). Significant differences were observed in the Bethesda cytopathology categories (*p* < 0.001), with the non-functioning group showing higher rates of indeterminate and suspicious/malignant classifications. Malignancy was more prevalent in non-functioning nodules compared to toxic adenomas (40.2% vs. 16.7%, *p* < 0.001). Among the 17 malignant toxic adenoma cases, papillary thyroid carcinoma (PTC) was the most frequent histological subtype (82.4%), and the malignancies were most commonly located in the right thyroid lobe (76.5%).

**Table 1:** Comparison of demographic and clinical characteristics between toxic adenomas and non-functioning nodule

Parameters	Toxic adenoma (*n* = 102)	Non- Functioning Nodule (*n* = 102)	*p*
Age (year)	52.40 ± 13.04	51.43 ± 10.42	0.590
Gender, F (n,%)	74 (72.5%)	73 (71.6%)	0.876
TSH (mIU/L)	0.05 (0.01–0.22)	1.35 (0.9–2.2)	< 0.001
Nodule size (mm)	32.41 ± 15.22	31.35 ± 13.64	0.605
Bethesda Classification (n,%) * • Bethesda I • Bethesda II • Bethesda III • Bethesda IV • Bethesda V • Bethesda VI	• 18 (22.8%) • 45 (57.0%) • 14 (14.7%) • 0 (0%) • 1 (1.3%) • 1 (1.3%)	• 24 (25.0%) • 14 (14.6%) • 38 (39.6%) • 4 (4.2%) • 8 (8.3%) • 8 (8.3%)	< 0.001
EU-TIRADS (n,%) • 2 • 3 • 4 • 5	• 1 (1%) • 89 (87.3%) • 9 (8.8%) • 3 (2.9%)	• 2 (2%) • 88 (86.3%) • 3 (2.9%) • 9 (8.8%)	0.077
Lymphocytic thyroiditis (%)	14.7%	21.6 %	0.201
Malignancy rate (n,%)	17 (16.7%)	41 (40.2%)	< 0.001
Pathologic diagnosis (n,%) • Papillary thyroid cancer • Follicular thyroid cancer • Medullary thyroid cancer	• 14 (82.4%) • 3 (17.6%) • 0 (0%)	• 37 (90.2%) • 2 (4.9%) • 2 (4.9%)	0.217
Tumor location (n,%) • Right lobe • Left lobe • Isthmus	• 13 (76.5%) • 4 (23.5%) • 0	• 14 (34.1%) • 24 (58.5%) • 3 (7.3%)	0.011

EU-TIRADS: European Thyroid Imaging and Reporting Data System, F: female; TSH, thyroid-stimulating hormone

*A total of 175 patients had available fine-needle aspiration biopsy (FNAB) data, including 79 patients with toxic adenomas and 96 patients with non-functioning thyroid nodules

A detailed comparison of malignant nodules in terms of histopathological features and clinical outcomes is presented in Table 2. Among malignant nodules, no significant differences were found between groups regarding tumor size, extrathyroidal extension, lymphovascular invasion, capsular invasion, multifocality, or lymph node metastasis (Table-2). The distribution of ATA risk categories and radioactive iodine therapy use was also similar. At 5-year follow-up, the rate of excellent response was high in both groups (88.2% vs. 97.4%, *p* = 0.216).

**Table 2:** Comparative analysis of pathological features and clinical outcomes of malignant nodules

Parameters	Toxic adenoma (*n* = 17)	Non- Functioning Nodule (*n* = 41)	*p*
Tumor size (mm)	8.5 (5.5–17.8)	14 (2.8–24)	0.887
Extrathyroidal extension (n,%)	0 (0%)	4 (9.8%)	0.310
Lymphovascular invasion (n,%)	1 (5.9%)	4 (9.8%)	0.632
Capsular invasion	3 (17.6%)	5 (12.2%)	0.681
Multifocality (n,%)	7 (41.2%)	19 (46.3%)	0.719
Lymph node metastases (n,%)	1 (5.9%)	7 (17.1%)	0.415
ATA risk stratification (n,%) * • Low • Low-intermediate • Intermediate-high • High	• 10 (58.8%) • 6 (35.3%) • 1 (5.9%) • 0 (0%)	• 15 (38.5%) • 16 (41.0%) • 6 (15.7%) • 2 (5.1%)	0.554
Radioactive iodine therapy (n,%)	8 (47.1%)	28 (68.3%)	0.129
Evidence of disease at 5-yr follow-up (n,%)* • Excellent response • Biochemical incomplete response • Structural incomplete response • Indeterminate response	• 15 (88.2%) • 0 (0%) • 1 (5.9%) • 1 (5.9%)	• 38 (97.4%) • 0 (7.2%) • 1 (2.6%) • 0 (15.9%)	0.216

ATA: American Thyroid Association yr: year

* Cases of medullary thyroid carcinoma were excluded from the final analysis due to their distinct histopathological and clinical characteristics.

**Fig. 2:**
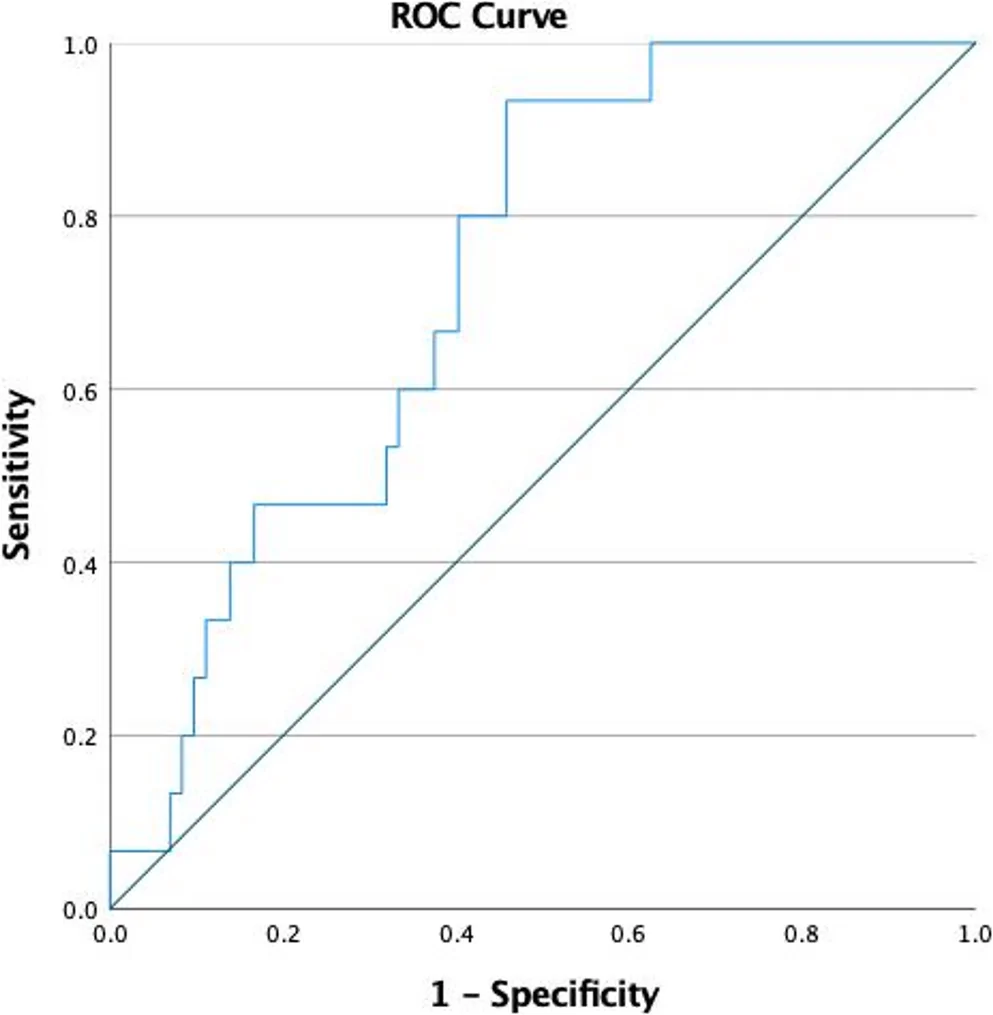
Receiver Operating Characteristic (ROC) Curve for the Ratio Parameter in Predicting Malignancy Among Toxic Thyroid Nodules. [The area under the curve (AUC) was 0.731 (95% CI: 0.614–0.847; *p* = 0.005). The optimal cut-off value was 2.58, yielding a sensitivity of 93.3% and a specificity of 54.2%.]

In patients diagnosed with toxic thyroid adenomas, a detailed comparison between malignant and benign nodules was conducted, as summarized in Table 3. In the subgroup analysis of patients with toxic adenomas, no significant differences were observed between malignant and benign cases in terms of age, gender, TSH, or nodule size (Table-3). However, patients with malignant toxic nodules had significantly higher fT4 levels (1.41 vs. 0.97, *p* < 0.001) and a higher fT4/fT3 ratio (2.48 vs. 2.80, *p* = 0.005). Additionally, malignant nodules more frequently exhibited higher EU-TIRADS categories (*p* = 0.039). To assess predictors of malignancy in toxic thyroid adenomas, both univariate and multivariate logistic regression analyses were conducted. In univariate analysis, both the fT4/fT3 ratio (B = 1.038, *p* = 0.011; OR = 2.82, 95% CI: 1.27–6.23) and high EU-TIRADS classification (grades 4–5 vs. 1–3) (B = 1.702, *p* = 0.012; OR = 5.49, 95% CI: 1.45–20.81) were found to be significantly associated with malignancy. A multivariate logistic regression analysis was performed to assess the independent predictive value of the fT4/fT3 ratio and EU-TIRADS classification for malignancy among patients with toxic thyroid adenomas. In the final model, the fT4/fT3 ratio was a significant independent predictor of malignancy (B = 0.926, *p* = 0.0027), with an odds ratio (OR) of 2.53 (95% CI: 1.11–5.73). In addition, patients with EU-TIRADS categories 4–5 had significantly greater odds of harboring malignancy compared to those with lower EU-TIRADS scores (B = 1.425, *p* = 0.049), with an OR of 4.16 (95% CI: 1.00–17.23). ROC curve analysis for the fT4/fT3 ratio in predicting malignancy among toxic nodules yielded an AUC of 0.731 (95% CI: 0.614–0.847; *p* = 0.005). A cut-off value of 2.58 provided a sensitivity of 93.3% and a specificity of 54.2% (Figure-2).

**Table 3:** Comparative analysis of malignant and benign nodules in patients with toxic thyroid adenomas

Parameters	Malign (*n* = 17)	Benign (*n* = 85)	*p*
Age (year)	52.18 ± 9.96	52.45 ± 13.63	0.938
Gender, F (n,%)	74 (72.5%)	73 (71.6%)	0.876
TSH (mIU/L)	0.01 (0.01–0.23)	0.06 (0.01–0.21)	0.296
fT4	1.41 (1.01–1.63)	0.97 (0.83–1.21)	< 0.001
fT3	4.38 (3.69–5.66)	4.05 (3.56–4.50)	0.194
fT4/fT3 ratio	2.80 (2.66–3.54)	2.48 (2.13–2.92)	0.005
Nodule size (mm)	28.59 ± 16.71	33.17 ± 14.89	0.259
EU-TIRADS (n,%) • 2 • 3 • 4 • 5	• 0 (0%) • 12 (70.6%) • 3 (17.6%) • 2 (11.8%)	• 1 (1.2%) • 77 (90.6%) • 6 (7.1%) • 1 (1.2%)	0.039
Lymphocytic thyroiditis (n,%)	4 (23.5%)	11 (12.9 % )	0.270

In 12 patients (11.8%), malignancy was detected in a lobe different from the one harboring the toxic adenoma. The mean age of this subgroup was 50.25 ± 11.9 years, and 9 (75%) were female. All malignancies were PTC, with a median tumor size of 6 mm (range: 5–9 mm). None of these incidentally discovered tumors exhibited lymphovascular invasion, extrathyroidal extension, capsular invasion, or lymph node metastasis. According to the ATA risk stratification system, one patient was classified as intermediate risk, while the remaining cases were considered low-risk.

## Discussion

In this surgically selected cohort, toxic adenomas demonstrated a 16.7% malignancy rate. Although this was lower than the 40.2% observed in surgically treated non-functioning nodules, it remains noteworthy within the operative population, particularly in the context of current guideline recommendations that often exclude hyperfunctioning nodules from cytologic evaluation 2, 3, 9. However, these findings reflect only surgically treated patients and should not be generalized to all individuals with hyperfunctioning nodules.

Although toxic adenomas exhibit autonomous function, they have traditionally been viewed as low-risk lesions in terms of malignancy, based on the suppressive effects of low serum TSH on thyroid follicular cell proliferation 1. However, accumulating evidence has begun to challenge this assumption in selected clinical settings. In the present study, conducted in a surgically selected cohort, malignancy was detected in 16.7% of operated toxic adenomas, a rate consistent with previously reported surgical series 4–6, 10. Although this proportion was significantly lower than the 40.2% observed among surgically treated non-functioning nodules (*p*< 0.001), it exceeds the sub-5% malignancy rates historically reported in earlier population-based studies[11, 12]. These findings suggest that the traditional perception of hyperfunctioning nodules as uniformly benign may not fully apply to patients selected for surgical management. Variations in thyroid cancer prevalence may be explained by differences in treatment strategies, surgical approaches, and the extent of histopathological examination across centers. Moreover, genetic and environmental factors, such as residence in endemic goiter regions and differences in iodine exposure, may also contribute to the variability in thyroid cancer rates. In our center, nodules with suspicious ultrasonographic features, regardless of etiology (Graves’ disease or autonomously functioning nodule), are routinely evaluated with FNAB once euthyroidism is achieved. This approach may have facilitated the detection of cytologically suspicious nodules and contributed to an increased rate of thyroidectomy decisions instead of long-term medical or radioactive iodine therapy.

Recent data reaffirm that PTC remains the predominant histologic subtype among malignancies detected within toxic adenomas 6, 10, 13. While earlier surgical series suggested notable rates of follicular or Hürthle cell carcinomas, more recent systematic reviews and cohort studies indicate that differentiated thyroid cancers—particularly PTC and its variants—account for the vast majority of malignancies in toxic nodules 4, 6, 10, 13, 14. Consistent with these findings, our study also demonstrated that PTC was the most frequently observed histological type among malignant toxic nodules.

The analysis of malignancy predictors in toxic adenomas yielded two key insights. First, the fT4/fT3 ratio was significantly higher among malignant cases, with a cut-off value of 2.58 achieving a sensitivity of 93.3%. This observation aligns with findings by Sasson et al., who reported that a T4/T3 ratio > 3.3 independently predicted differentiated thyroid cancer (OR: 4.9; *p* < 0.01) 15. A plausible explanation is impaired type 1 and type 2 deiodinase activity, which reduces the conversion of fT4 to active fT3, resulting in an elevated fT4/fT3 ratio 16, 17. This enzymatic dysfunction may stem from altered expression of regulatory transcription factors such as Titf1/Nkx2-1 and Pax-8, frequently disrupted in malignant tissue 18. Furthermore, tumor dedifferentiation leads to the loss of thyroid-specific gene expression involved in iodine metabolism and hormone activation, further exacerbating the imbalance between fT4 and fT3 [19]. These molecular alterations may underlie the biochemical profile observed in thyroid malignancies and support the potential utility of the fT4/fT3 ratio as a surrogate oncologic marker. The current study indicate that an elevated fT4/fT3 ratio may serve as a novel biochemical marker for increased malignancy risk in toxic adenomas. Although the marker demonstrates high sensitivity, its relatively low specificity limits its utility as a standalone diagnostic tool. Therefore, further prospective studies incorporating molecular profiling are needed to validate its predictive accuracy and to enhance individualized risk stratification in patients with toxic thyroid nodules.

Second, the present analysis demonstrates the diagnostic value of the EU-TIRADS classification system in evaluating malignancy risk within toxic adenoma. Despite their traditionally benign classification, toxic adenomas rated as EU-TIRADS 4–5 showed a significantly higher malignancy risk, and this remained an independent predictor in multivariate analysis, highlighting the clinical relevance of ultrasound even under TSH suppression. Supporting this, recent studies have demonstrated that suspicious ultrasonographic risk markers retain predictive utility in isolated toxic adenomas. Chang et al. found that approximately 36.3% of scintigraphically hot nodules displayed at least one suspicious ultrasound feature according to ATA criteria, and these nodules were more likely to undergo biopsy or surgical excision despite their hyperfunctioning status 20. Mirfakhraee et al. reported that 36% of scintigraphically hot nodules demonstrated at least one high-risk ultrasound feature, and despite their functional autonomy, these nodules were associated with a higher-than-expected rate of malignancy 14. This emphasizes the need to assess sonographic risk features and biochemical patterns in tandem, rather than relying solely on scintigraphic findings.

Another critical finding in our study was that 11.8% of malignancies were incidental thyroid carcinomas. These lesions were predominantly papillary thyroid microcarcinomas with indolent histological features. Recent evidence suggests that the risk of incidental thyroid carcinoma in toxic nodular thyroid disease is significantly higher than once assumed 19–21. A multicenter study reported incidental malignancy in 5.9% of patients with toxic multinodular goiter and 4.8% of those with toxic adenoma, while a recent single-center series documented malignancy in approximately 22% of patients undergoing surgery for toxic goiter 21, 22. Similarly, in a large retrospective cohort of hyperthyroid patients undergoing total thyroidectomy, Berker et al. reported a 5.4% prevalence of incidental thyroid carcinoma in subcentimeter nodules, all of which were papillary microcarcinomas 23. Dirikoç et al. reported a 15.3% prevalence of incidental thyroid carcinoma in patients with toxic adenoma, with the vast majority of cases being PTC 6. These findings highlight the need for clinical vigilance and thorough preoperative assessment. Moreover, the presence of cancer in the contralateral lobe, unrelated to the toxic nodule, emphasizes the need for a comprehensive assessment of the whole thyroid, not just the hyperfunctioning region.

There is no clear evidence indicating that thyroid carcinomas arising from toxic adenomas exhibit more aggressive behavior compared to those originating from non-toxic nodules 5, 6, 13. Comparative studies found no significant differences in tumor size, invasion, or ATA risk classification between toxic and non-toxic nodules 5, 13. Although rare cases of aggressive behavior have been reported in toxic adenoma, most cancers, regardless of functional status, are papillary microcarcinomas with indolent features 24–26. Consistent with these findings, our study also demonstrated no significant difference in histopathological characteristics or 5-year response rates between the two groups, suggesting that carcinomas from toxic nodules follow a comparable clinical course and should not be considered inherently less or more aggressive. To our knowledge, this is the first study to report long-term follow-up data comparing oncologic outcomes of thyroid carcinomas originating from toxic versus non-toxic nodules.

This study has several limitations. First, its retrospective design and inclusion of only surgically treated patients may introduce selection bias. Second, molecular profiling (e.g., BRAF, RAS mutations) was not available, limiting insight into the genetic underpinnings of malignancy. Third, the relatively small number of malignant toxic adenomas (*n* = 17) may restrict the power of multivariate analysis. Additionally, the interpretation of ultrasound features and subsequent EU-TIRADS classification may be subject to interobserver variability, which could influence risk stratification and limit the generalizability of the current data. Finally, the follow-up period, although extending to five years, may be insufficient to detect late recurrence in some low-risk cases. Despite its limitations, this study has multiple strengths. It is one of the few to compare toxic and non-toxic nodules using a propensity score-matched design, thereby minimizing confounding related to age, sex, and nodule characteristics. The study introduces the fT4/fT3 ratio as a novel and accessible biochemical marker for malignancy risk. It also provides insights into contralateral lobe malignancies in patients with toxic adenomas, which carry important implications for both surgical planning and postoperative surveillance. Moreover, the inclusion of detailed histopathologic characteristics, ATA risk stratification, and long-term oncologic outcomes enhances the robustness of the analysis and adds clinical relevance to the study’s conclusions.

In conclusion, contrary to traditional expectations, findings from this surgically selected cohort demonstrate that toxic adenomas are not invariably benign. Although scintigraphic hyperfunction is traditionally considered reassuring, our findings indicate that biochemical profiles, ultrasonographic features, and a comprehensive assessment of the entire thyroid gland may still hold diagnostic relevance in patients selected for surgery. These observations apply exclusively to individuals undergoing operative management and should not be extrapolated to the broader population of patients with hyperfunctioning nodules. Further prospective and population-based studies are warranted to define the true prevalence of malignancy in toxic adenomas and to refine evidence-based risk stratification strategies.
